# Supplementary material for: Topologically trivial gap-filling in superconducting Fe(Se,Te) by one-dimensional defects
Source: Nat Commun. 2024 May 6;15:3774. doi: 10.1038/s41467-024-48047-0 (PMC11074306; doi:10.1038/s41467-024-48047-0)
Supplement: Supplementary file 1 — Supplementary Information [file 41467_2024_48047_MOESM1_ESM.pdf]

Supplementary Information to 'Topologically trivial gap-filling in superconducting Fe(Se,Te) by one dimensional defects'

A. Mesaros[1], G. D. Gu[2] & F. Massee[1]

[1]*Université Paris-Saclay, CNRS, Laboratoire de Physique des Solides, 91405, Orsay, France*
[2]*Condensed Matter Physics and Materials Science Department, Brookhaven National Laboratory, Upton, NY 11973, USA*

1 Integer $\mathbf{K}_\alpha$, arbitrary $\mathbf{K}_\alpha$ and real-space fitting analysis

Throughout this work the phase shifts in the atomic lattice are extracted using Fourier transform analysis. We refer to Ref. [1] for a detailed description of the analysis technique. We stress that we use the exact same technique as this work and e.g. Ref. [2], but extend the analysis to include non-integer pixel values of the reference lattice Bragg vector. This is essential because the experimental pixel size is generally not commensurate with the atomic lattice, hence a Bragg peak is in practice never at integer pixel coordinates. The optimization method we introduce based on minimizing the slope term in the phase field recovers an optimal (fractional) coordinate of the reference lattice vector, $\mathbf{K}_\alpha$. Fig. S1 demonstrates the difference in the extracted phase fields when the optimal $\mathbf{K}_\alpha$ and the closest integer-valued $\mathbf{K}_\alpha$ are compared. The histogram of phase values clearly shows that the optimal (fractionally-valued) $\mathbf{K}_\alpha$ reveals quite homogeneous domains and the rigid shift between them, while the closest integer-valued $\mathbf{K}_\alpha$ introduce large unphysical spatial fluctuations. We note that the histograms only consider the parts of the image where the atomic contrast is not obscured by the 1D defect (see Fig. S1i). The distance between the respective peaks therefore gives an accurate value for the phase shift across the 1D defect and is used throughout this work.

Another technique introduced by Ref. [3] obtains phase information through a real-space fitting routine. To ensure that our Fourier-transform approach does not introduce artefacts or systematic errors, we have applied this technique as well to several of our topographies. Since the real-space method calculates the phase fields but does not determine $\mathbf{K}_\alpha$, we input the optimal values of $\mathbf{K}_\alpha$ which we obtained from our Fourier transform analysis. After applying the same Gaussian filtering window as in the Fourier transform analysis, and then inverse Fourier transforming, we obtain the two real-space images (one for each Bragg peak) that will be used for fitting. For the real-space fit we select a sliding window of roughly one wavelength, and use a roughly 50% overlap between subsequent windows. We note that similarly to Ref. [3] the exact size of the window does not significantly influence the outcome of the fit as long as it is not much smaller or much larger than one wavelength. As the comparison in Figs. S1g,h shows, both methods give identical results, which is strong support for the validity of our analysis. The main drawback of the

real-space fitting method is that it is very time-consuming since a large number of real-space fits are required, of which some do not easily converge due to e.g. limited local contrast.

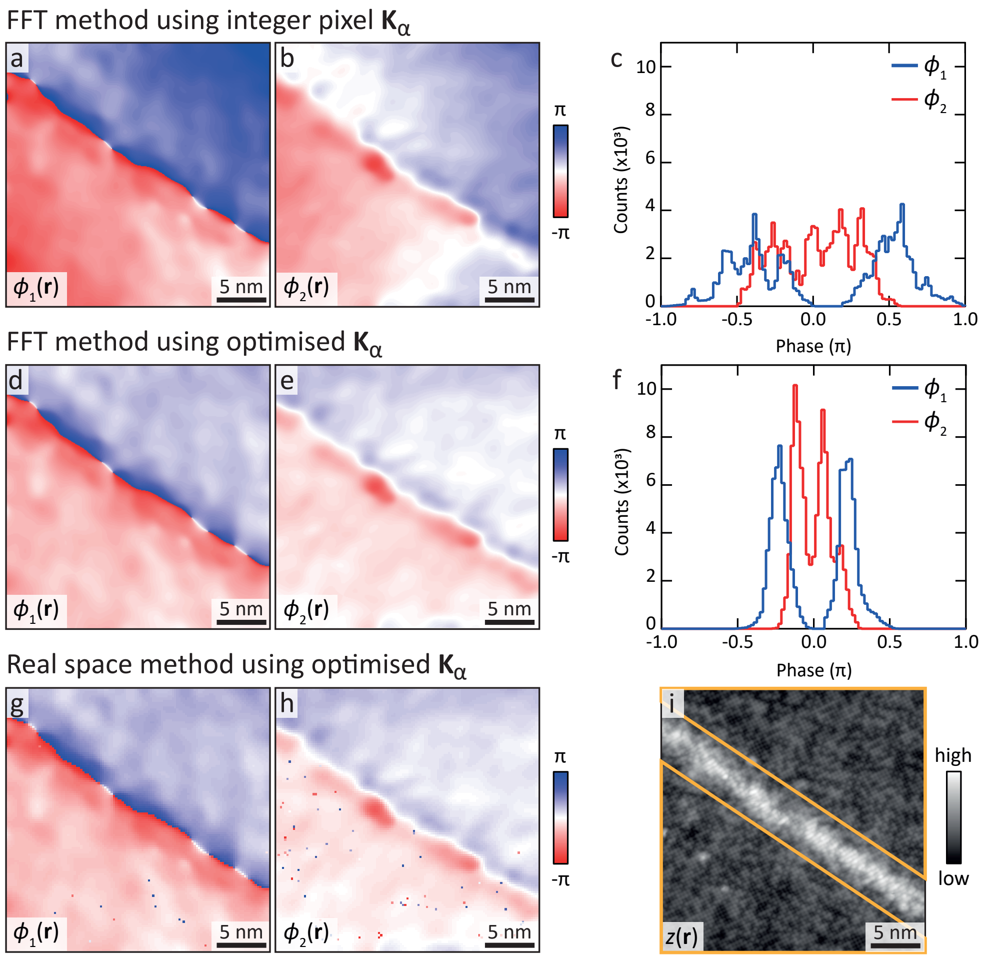

Figure S1 Comparison of techniques. a, b Phase images for the same 1D defect as main text Fig. 2b but using an integer pixel value for the $\mathbf{K}_\alpha$: (K_{1x}, K_{1y}) = (245, 150), (K_{2x}, K_{2y}) = (153, 120). **c** Histograms of **a**, **b** considering only the regions where the atomic contrast is not obscured by the 1D defect (see below). **d-f** Same as **a-c**, but using the optimal $\mathbf{K}_\alpha$: (K_{1x}, K_{1y}) = (244.65, 150.27), (K_{2x}, K_{2y}) = (152.67, 120.0). In this case, there is no slope in the phase images and the phase shifts across the 1D defect can be accurately extracted from the histograms. **g, h** Phase images determined using the real-space fitting method with the optimal $\mathbf{K}_\alpha$. The results are identical to **d**, **e** except for a few pixels where the real-space fit did not converge properly. **i** Constant current image that was used to extract the phase images in this figure. The orange lines mark the regions considered for the histogram and phase slope minimization, i.e. the area between the lines where the 1D defect is located is not considered as the atomic contrast is obscured here.

2 Phase shift in presence of non-linear strain or drift

In this work we are purely interested in the phase jump across the 1D defect and would like to avoid or compensate for possible additional position-dependent changes in the phase. In principle, the lattice in absence of defects has a single lattice constant. We have confirmed this on a number of topographs taken far from 1D defects. Since there is no reason for either side of the 1D defect to be different, we can safely assume that the 1D defect only locally distorts the phase. To determine the optimal lattice parameter, we therefore combine the standard deviation of both sides of the 1D defect. In most cases, using only one of the sides of the 1D defect to determine the reference lattice will give near-identical results (see Fig. S2g-j).

One may wonder, however, if there are changes in the phase possible that could affect the reliability of our phase jump extraction method, or where perhaps only one side of the 1D defect should be considered. Since we optimize the reference lattice parameter, only non-linear changes to the lattice constant are important: a linear compression or expansion will merely produce a slightly different reference lattice parameter, but still a constant phase across an image (and thus reliable jump). There are two possibilities for non-linearity: drift and strain. Drift, due to the slow (temperature dependent) relaxation of the piezos after a change in voltage, will stretch or compress an image in the direction of the drift. In this work we have tried to minimize non-linear drift by staying in the same location for extended periods of time, waiting sufficiently long after a temperature change and scanning slowly. Nevertheless, non-linear drift could still appear, for example in the first image after a temperature change. This, however, always shows up at the start of the image and in the slow scan direction (the y-axis in all our data) and as such can easily be recognized. For vertically running 1D defects, using both sides of the 1D defect is therefore valid this case. Nevertheless, whenever we did observe non-linear drift, we have not taken the affected part of the image into consideration.

A more interesting case is when the lattice constant itself is changing non-linearly as function of position due to strain. Again we stress that a linear strain field will be corrected for by our optimization method. A non-linear strain field, however, will give a different slope of the phase for the left and right region of the image. Let us consider a situation where the lattice parameter is constant on the left, has a positive phase jump to the right and then progressively compresses on the right (other configurations will lead to the same conclusion). Optimizing the reference lattice using the left side only will give a constant phase on the left, a positive jump followed by a phase with a negative slope on the right. Clearly, this will lead to an underestimation of the jump in the phase, as the negative slope partially counteracts the positive jump. Conversely, if the lattice is optimized on the right side only, the jump will be overestimated. Figure S2b-e shows an example of exactly such a situation. Here, the 1D defect is close to a large scale depression in topography, likely associated with strain in the sample. As the analysis using either the left side, right side or both shows, the most reliable phase jump is extracted when taking both sides into account, as we do throughout this work. Simple modelling of non-linear strain fields confirms this conclusion.

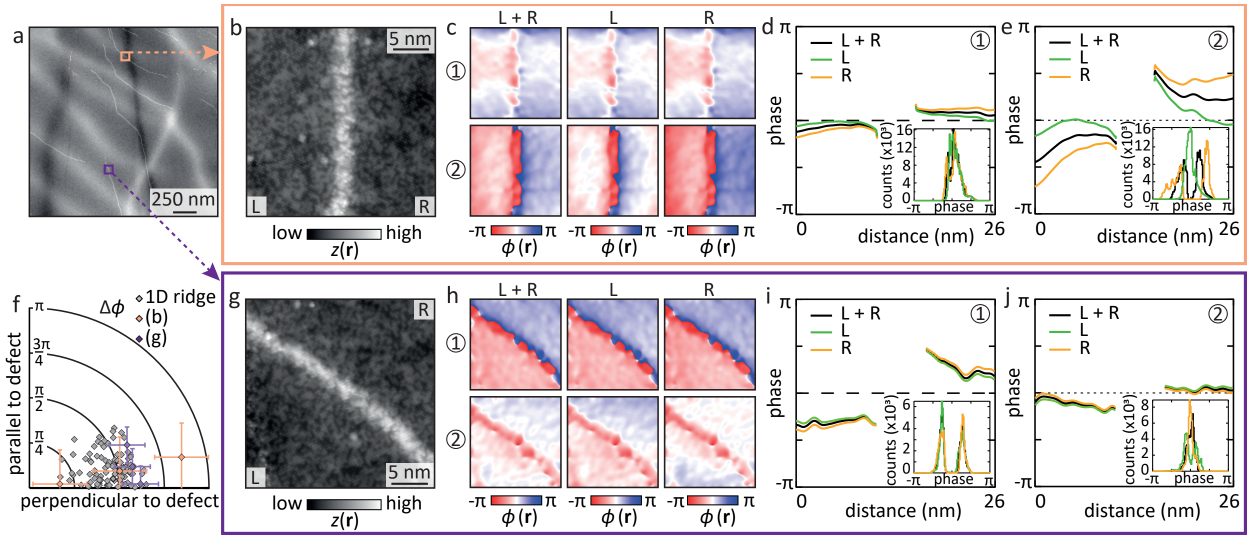

Figure S2 Phase analysis with(out) strain. **a** Large field of view (same as main text Fig. 1a). **b** Constant current image of 1D defect nearby the large scale dark depression in panel a, which is likely due to strain. **c** Phase analysis for the two lattice vector directions (1 and 2) upon optimising the reference lattice using both sides of the defect (L+R), or either the left (L) or right (R) side only. **d, e** Horizontal line-cut through the middle of the phase images in panel c for lattice vectors 1 and 2, respectively. **f** Phase jump and its direction with respect to the defect for the three cases (L+R, L and R, orange markers). To avoid under- or overestimating the phase jump in presence of strain, both sides need to be taken into account. **g-j** Same as b-e on a region without strain. The resulting phase jump magnitude for the three cases is near identical (purple markers in f).

To best clarify the interplay of our phase-optimization approach and the physical quantities such as phase-jump and non-linear strain, in Fig. S2 we include error bars on the extracted values of the phase jump. These error bars represent the statistical error, obtained in principle by adding in quadrature two relative errors: one from the spatial variation of the phase field (an error quantified by standard deviation of the phase field across both regions), and the other from optimizing $\mathbf{K}_\alpha$ (quantified by the fit-error in finding the minimum value of $\varepsilon_{\phi\alpha}(\mathbf{K}_\alpha)$), the latter being negligibly small throughout this work. These error bars include some of the uncertainty due to the spatial fluctuations of strain, but they do not at all contain the systematic error made by the method one chooses to remove the underlying smooth non-linear strain profile. As the example in the bottom row of Fig. S2 demonstrates, when we extract the phase jump using three methods, namely, considering only the R region, only the L region, or both, we get three values of phase jump magnitude which agree within the statistical error bars. This is strong indication that non-linear strain is negligible in this example, i.e. we do not have a systematic error due to strain. The example in top row of Fig. S2 produces three values of phase jump that do not overlap within the statistical error bars. Hence, we know that the three methods (R only, L only, or both) give three distinguishable reference lattice which compensate differently for the non-linear strain. As argued in previous paragraph, the method using both L and R will minimize the bias in phase jump, and gives a value

in between the ones obtained from only L or only R. Hence, for the few fields-of-view which are near a strained area, e.g. top row of Fig. S2, we report the result of our usual method that uses both L and R.

Finally, it is worth noting that using a reference lattice which is strictly integer-valued in pixels of the Fourier space is simply a systematic error which is more problematic, since it affects all phase jumps (not only near heavily strained areas), and may drastically bias the phase jump (able to move it from $\sim\pi/2$ to π) simply because a shift of $\mathbf{K}_\alpha$ by half a pixel corresponds to a spurious accumulation of phase of order π across the field of view that may easily bias the extracted phase jump at the defect (see the schematic in main text Fig. 2a).

3 Dip-like 1D defect

In addition to the ridge-like 1D defect that is the focus of the main text, we found another 1D defect that appears as a depression in topography (i.e. it is dip-like). Figure S3a shows a segment of this defect. Unlike the ridge-like defect, all atoms are clearly visible in the dip-like defect, as evidenced from the enlargement in Fig. S3b. As the guides to the eye highlight, there is a clear shift in the atomic lattice in the vertical direction upon crossing the defect. Using the same phase analysis as for the ridge-like defect, we extract the phase shift in the direction of the two Bragg peaks, see Figs. S3c,d. The magnitude and angle with respect to the defect for several locations along the hundreds of nm long defect are plotted in main text Fig. 2f: the magnitude is similar to that of the ridge-like defect ($\sim\pi/2$), but the shift direction is more along the defect instead of perpendicular to it as is the case for the ridge-like defect.

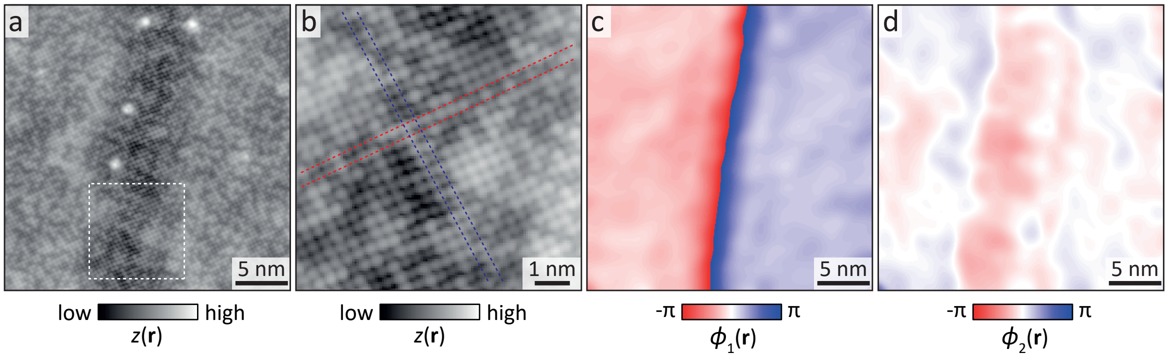

Figure S3 Dip-like defect: phase. a Constant current image of a segment of the dip-like 1D defect, the dip is several nm wide and runs near-vertical. Setup: V = 5 mV, I = 50 pA. **b** Enlargement of the dashed box in panel **a** showing clear atomic contrast at the defect. The red and blue dashed lines are guides to the eye highlighting the shift of the atoms in the vertical direction upon crossing the defect. **c**, **d** Phase images for the near vertical and near horizontal Bragg peaks, respectively. The total magnitude of the phase shift is $\Delta\phi$ = 0.46$\pi \pm$ 0.1π.

Since the dip-like defect also generates a phase shift of the lattice, which is of the same order of magnitude as that for the ridge-like defect, the question is whether there is any signature in differential conductance that would suggest the presence of topological non-trivial states. Figure 4 shows two examples that illustrate the absence of such signatures. The zero bias conductance at the endpoint of the dip is only showing intensity at excess iron impurities, with little to no contribution from the 1D defect. The marked contrast between the zero bias conductance of the dip-like defect and the ridge-like defect can be seen in the crossing of the two in Figs. 4c,d: whereas a strong sub-gap intensity is apparent on the latter, the former is barely, if at all, visible.

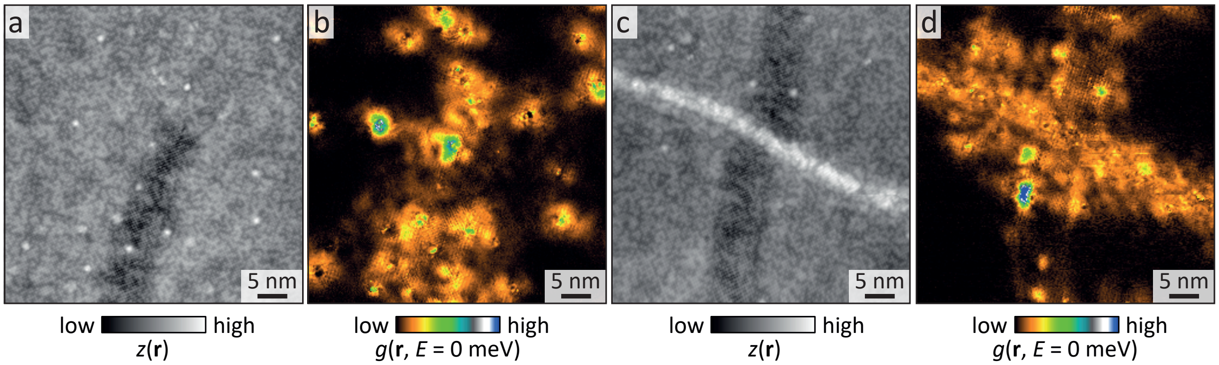

Figure S4 Dip-like defect: conductance a Constant current and **b** zero bias conductance at the endpoint of the dip-like 1D defect. **c**, **d** Same as panels **a**, **b**, but for a location where the dip-like defect crosses a ridge-like defect. Despite a similar phase shift of the lattice, the dip-like 1D defect shows hardly any sub-gap conductance, if at all. Setup conditions for both measurements: $V = 5$ mV, $I = 100$ pA

4 Surface debris

Main text Figs. 3a,b show a region where a long 1D defect is broken up: two internal endpoints are seen some tens of nanometres apart. Interestingly, in between the endpoints a strip of debris is located. As can be seen in the full field of view data in Fig. S5, a second, smaller strip of debris is located a little farther from the gap in the 1D defect. If the two strips of debris are laid end-to-end, they are not far from bridging the gap between the two internal endpoints, almost suggesting they once did, but were somehow ejected. The striking similarity in both shape (height, width and one-dimensionality) and sub-gap filling strongly suggests the two objects are actually the same: in one case residing on top of the surface (the debris, causing no lattice shift) and in the other case below the top layer (the ridge-like 1D defect, causing a lattice shift in the warped top layer).

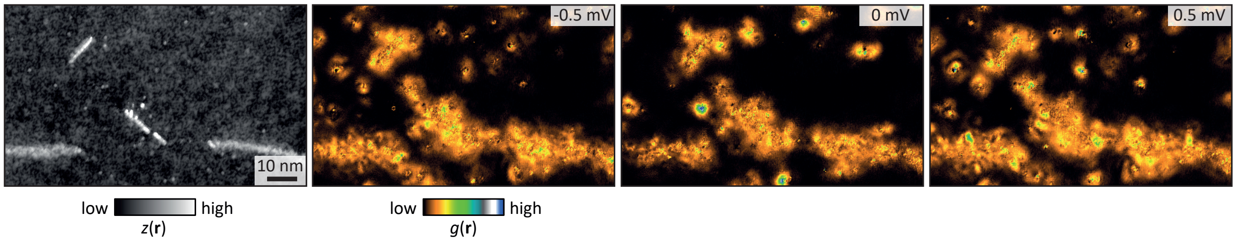

Figure S5 Gaps and debris. a Constant current image and **b**-**d** simultaneously recorded differential conductance at three different voltages (-0.5 mV, 0 mV, 0.5 mV). Both at zero bias and at other sub-gap voltages the 1D defect and surface debris are nearly indistinguishable in differential conductance. For the surface debris, no phase shift of the lattice is present. Main text Figs. 3a and b are the lower parts of the topography and zero bias conductance shown here, respectively. Setup: V = 5 mV, I = 100 pA.

To highlight that the sub-gap filling along the ridge-like 1D defects is not unique to these objects, Fig. S6 shows another example of surface debris where in absence of a phase shift of the lattice a near identical gap filling is observed (see e.g. Fig. S10 and S11 for comparison).

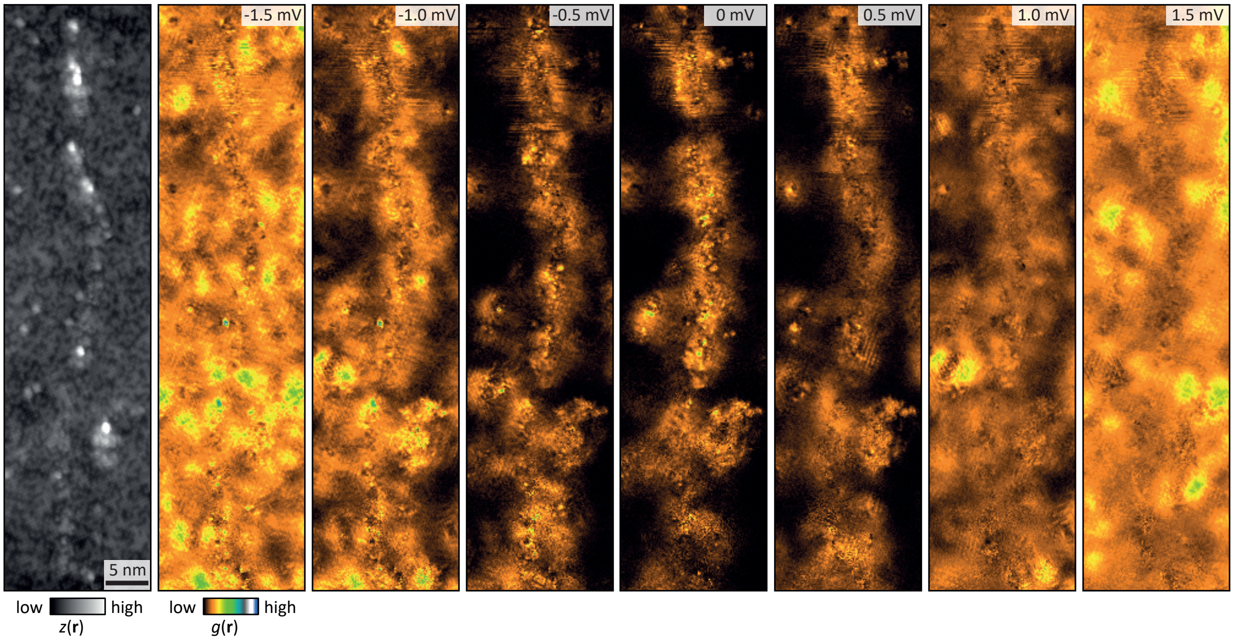

Figure S6 Surface debris: gap filling. Constant current image (left) and simultaneously recorded differential conductance (right) ranging from the negative to positive gap edge of a string of surface debris. Setup: V = 5 mV, I = 80 pA. The gap filling is near identical to that of the ridge-like 1D defects, yet is clearly of topologically trivial origin as the debris is disconnected and is not accompanied by a phase shift of the lattice.

5 Conductance gaps along the 1D defect

Main text Figs. 3d,e shows a location on one of the ridge-like 1D defects where a break in the sub-gap differential conductance is observed. Although such locations are rare, we found several other cases. Figs. S7a,b show one example where the hundreds-of-nanometres-long 1D defect is continuous, yet around a certain location a gap is recovered in the differential conductance. The enlargement of this area in Figs. S7c,d illustrates that the 1D defect itself does not look different in topography, whereas the zero-bias conductance is clearly vanishing due to a recovery of the gap in the density of states. Another example is shown in Figs. S7e,f where two 1D defects are running parallel to each other and looking the same, while one of them has two short segments in which the zero-bias conductance vanishes. In all cases, the phase shift of the lattice across these segments does not differ from the rest of the 1D defect. This behaviour is not compatible with topologically protected modes.

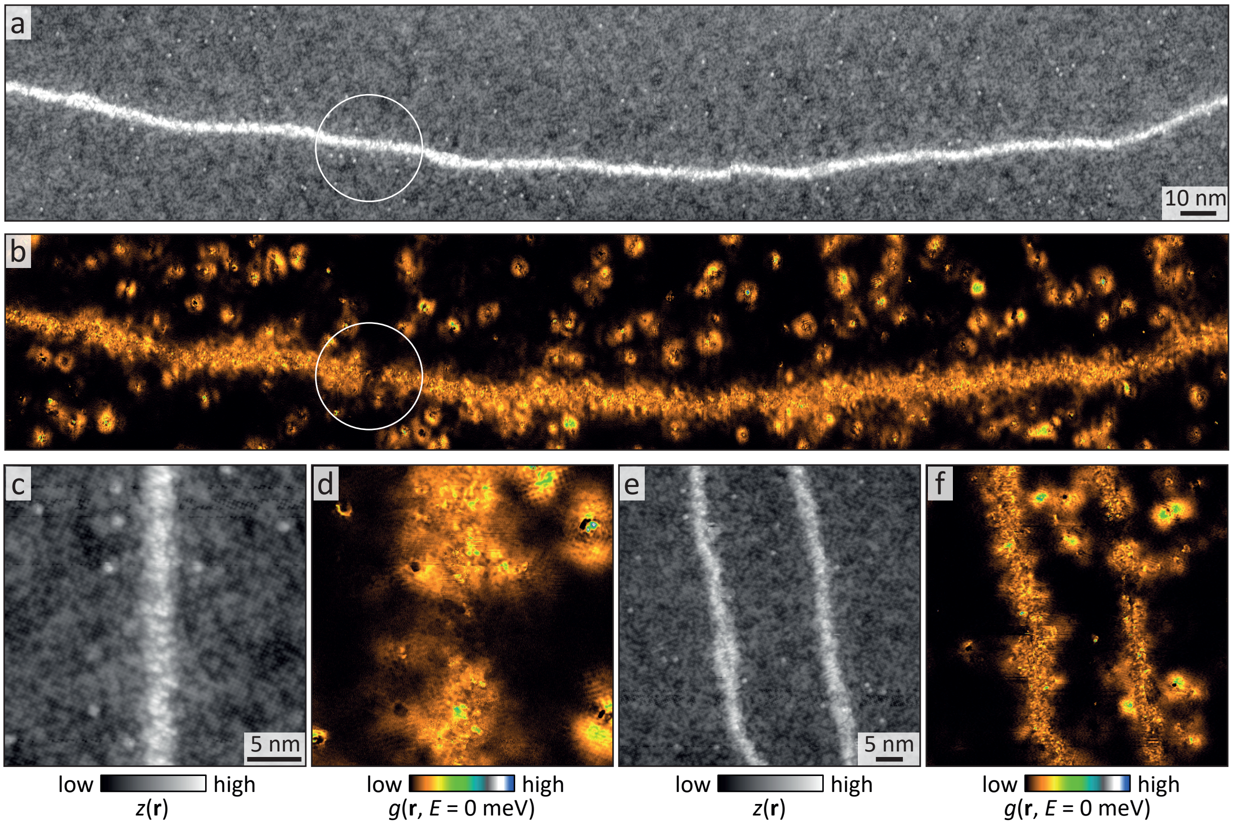

Figure S7 Gaps in zero bias conductance. a Constant current and **b** zero bias conductance of a large stretch of ridge-like 1D defect. Whereas the zero bias conductance is enhanced along the majority of the defect, a small region (indicated by the circle) is not. **c**, **d** enlargement of the gapped region in panels **a**, **b** respectively. **e**, **f** Another example of gapping on the 1D defect, in this case on one of two 1D defects that run parallel to each other (right-top). For all panels the setup conditions are V = 5 mV, I = 100 pA.

6 Manipulation

As was shown in main text Fig. 4, we managed to move one of the 1D defects. Although we have observed instabilities on multiple 1D defects (at moderate junction resistances, $\sim$50 MΩ), in all cases the defect snapped back to its original location. Two examples of such a temporary displacement are shown in Fig. S8. These measurement clearly show that only the 1D defect is manipulated, without affecting the tip or surface: the lattice is continuous across the line where the 1D defect snaps back. We note that even for high junction resistances often spectra taken on 1D defects (as well as on surface debris) show enhanced noise with respect to those taken on defect free areas, as exemplified by the spectra in main text Fig. 3c. This is directly related to the local instability of the defects or sections thereof to the presence of the tip. The case of Fig. 4 was the only instance where the manipulation of a large section of 1D defect was permanent: it did not snap back again and remained in its new location for the remainder of our study. Figure S9 shows a larger field of view measurement before and after manipulation of the same 1D defect as that shown in Fig. 4. To achieve this permanent manipulation, we scanned the tip at a high speed (120 nm/s versus our usual speed of $\sim$8 nm/s) and at a relatively low junction resistance (2.5 MΩ). Of course at such settings there is unfortunately always a risk of altering the tip and/or surface as well. In this case, one excess Fe atom was inadvertently picked up by the tip (and later dropped elsewhere again): the atomic structure, including the large hole and all other excess Fe atoms were not affected by the manipulation.

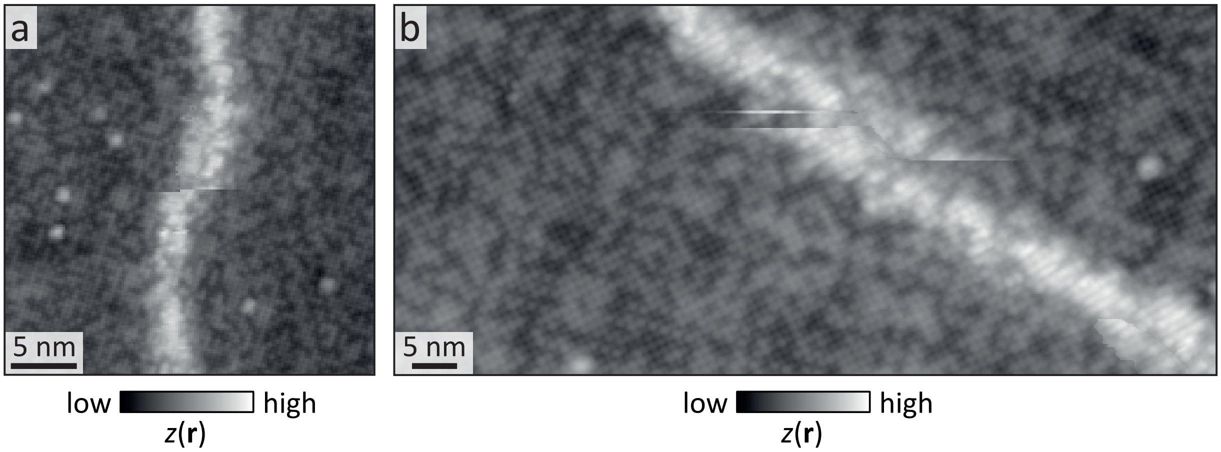

Figure S8 Temporarily moving 1D defect. **a**, **b** Constant current images on two different 1D defects. During imaging, both 1D defects move sideways and suddenly jump back. The atomic lattice is continuous across the line where the 1D defect jumps, showing that only the 1D defect is moved while the tip and surface remain the same. The y-axis is the slow scanning direction. Setup for both images: V = 5 mV, I = 100 pA.

As further discussed in the next section, the differential conductance at the endpoint of the 1D defect (top of Fig. S9) is unremarkable. More importantly, in addition to enhanced sub-gap

density of states on the ridge-like 1D defect, the differential conductance is also enhanced on another section that does not have a ridge-like 1D defect, forming an inverted Y in Fig. S9b. This section does not show a phase shift of the lattice, but actually appears damaged: atoms are missing and mixed in with point defects (see also main text Fig. 4a). It is most likely this damage that manages to pin the 1D defect in place once the electric field of the tip has dislocated it from its original position. In a series of measurements, we were indeed able to shift the 1D defect step-by-step, each time snapping a new section to the line of damage. This shows that the 1D defect is a flexible, movable object, not unlike the surface debris.

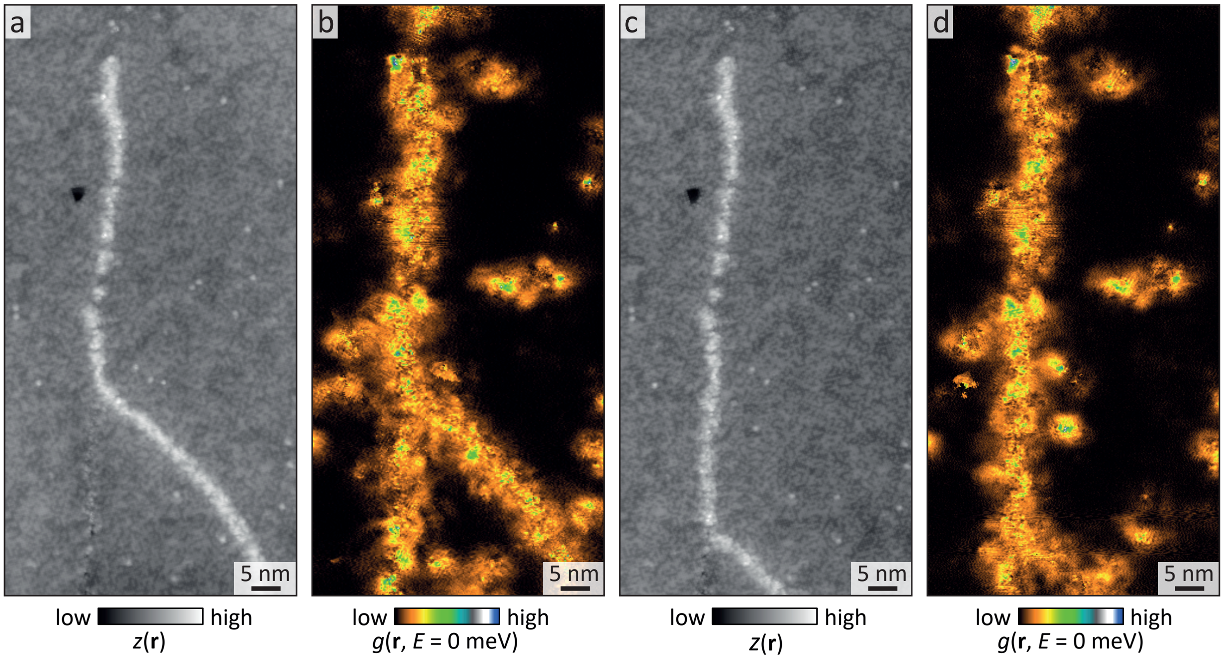

Figure S9 Permanently moving 1D defect. **a** Constant current image and **b** simultaneously recorded zero bias conductance before manipulation. **c**, **d** The same as panels **a**, **b**, respectively, after manipulation. Setup: V = 5 mV, I = 80 pA.

7 End points

As discussed in the main text, even though based on our analysis the 1D defects do not host topologically non-trivial dispersing modes, their end-points may still host non-trivial modes, similar to helical spin chains on a superconductor. As main text Figs. 3a,b show, however, there does not appear to be a remarkable change in density of states at the ends of the defects. To illustrate this in more detail, Figs. S10 and S11 show the evolution of the differential conductance on the endpoints of two other 1D defects. Neither the end of a long defect (Fig. S10, hundreds of nm) or both ends of a relatively short one (Fig. S11, $\sim$80 nm) are different from the midsection of the defect. Note also the striking resemblance of either data-set to that of the string of debris in Fig. S6.

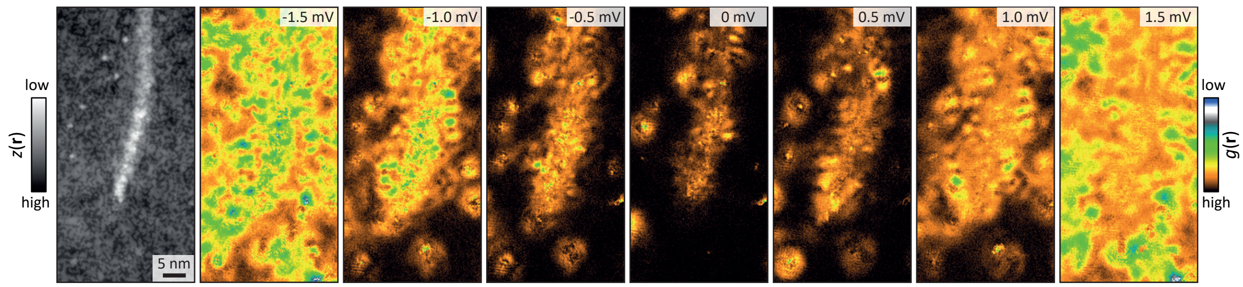

Figure S10 Constant current image (left) and simultaneously recorded differential conductance (right) ranging from the negative to positive gap edge of an endpoint of a 1D defect that is hundreds of nm long. Setup: V = 5 mV, I = 100 pA.

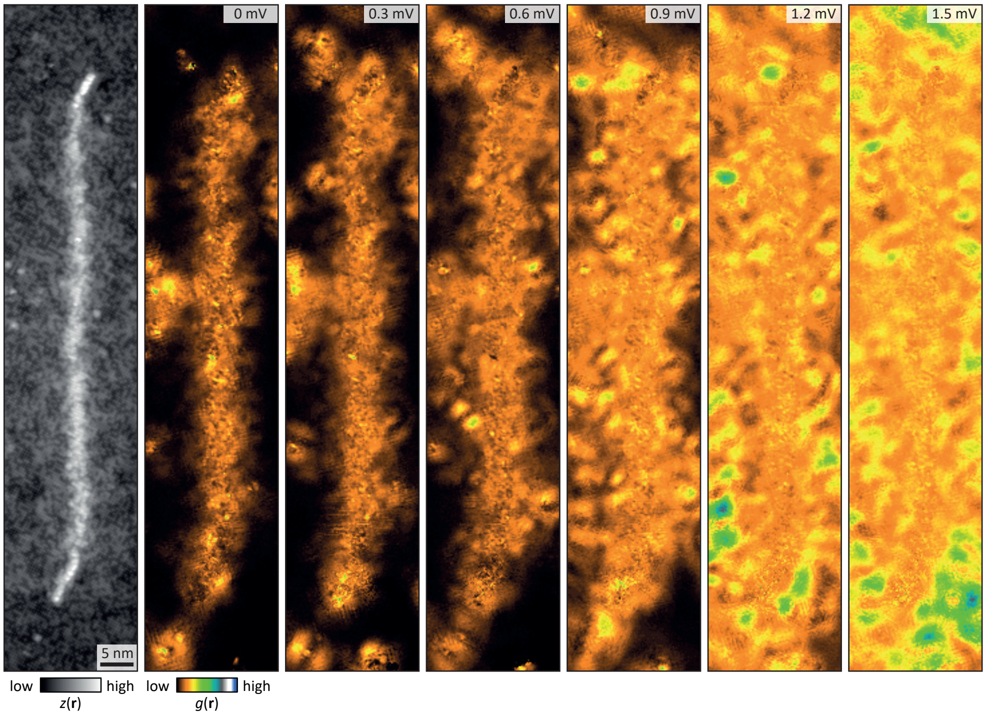

Figure S11 Constant current image (left) and simultaneously recorded differential conductance (right) ranging from zero bias to the positive gap edge of a relatively short 1D defect (∼80 nm). Setup: V = 5 mV, I = 100 pA.

References

1. M. J. Lawler, K. Fujita, J. Lee, A. R. Schmidt, Y. Kohsaka, C. K. Kim, H. Eisaki, S. Uchida, J. C. Davis, J. P. Sethna and E. -A. Kim, Nature **466**, 347–351 (2010) *Intra-unit-cell electronic nematicity of the high-T_c copper-oxide pseudogap states*

2. Z. Wang, J. Olivares Rodriguez, L. Jiao, S. Howard, M. Graham, G. D. Gu, T. L. Hughes, D. K. Morr and V. Madhavan, Science **367**, 104-108 (2020) *Evidence for dispersing 1D Majorana channels in an iron-based superconductor*

3. Á. Pásztor, A. Scarfato, M. Spera, C. Barreteau, E. Giannini and Ch. Renner, Phys. Rev. Research **1**, 033114 (2019) *Holographic imaging of the complex charge density wave order parameter*
